# Implementation of machine learning algorithms to create diabetic patient re-admission profiles

**DOI:** 10.1186/s12911-019-0990-x

**Published:** 2019-12-12

**Authors:** Mohamed Alloghani, Ahmed Aljaaf, Abir Hussain, Thar Baker, Jamila Mustafina, Dhiya Al-Jumeily, Mohammed Khalaf

**Affiliations:** 1 The Artificial Intelligence Department-, Dubai, UAE; 2 Liverpool John Moores University, Liverpool, UAE; 3 The University of Anbar, Al-Tameem Street, Al-Anbar, Al-Ramadi, 55431 Iraq; 4 Kazan Federal University, Kremlyovskaya St, Kazan, Republic of Tatarstan, 420008 Russia; 5 Department of Computer Science, Al-Maarif University College, Anbar, The city of Ramadi, 31001 Iraq

**Keywords:** Machine learning, Linear discriminant, Algorithms, Support vector machine, Diabetes re-admission, HbA1c

## Abstract

**Background:**

Machine learning is a branch of Artificial Intelligence that is concerned with the design and development of algorithms, and it enables today’s computers to have the property of learning. Machine learning is gradually growing and becoming a critical approach in many domains such as health, education, and business.

**Methods:**

In this paper, we applied machine learning to the diabetes dataset with the aim of recognizing patterns and combinations of factors that characterizes or explain re-admission among diabetes patients. The classifiers used include Linear Discriminant Analysis, Random Forest, k–Nearest Neighbor, Naïve Bayes, J48 and Support vector machine.

**Results:**

Of the 100,000 cases, 78,363 were diabetic and over 47% were readmitted.Based on the classes that models produced, diabetic patients who are more likely to be readmitted are either women, or Caucasians, or outpatients, or those who undergo less rigorous lab procedures, treatment procedures, or those who receive less medication, and are thus discharged without proper improvements or administration of insulin despite having been tested positive for HbA1c.

**Conclusion:**

Diabetic patients who do not undergo vigorous lab assessments, diagnosis, medications are more likely to be readmitted when discharged without improvements and without receiving insulin administration, especially if they are women, Caucasians, or both.

## Introduction

The approaches used in managing maladies have a major influence on the medical outcome of the patient including the probability of re-admission. A growing number of publications suggest the urgent needs to explore and identify the contributing factors that imply critical roles in human diseases. This can help to uncover the mechanisms underlying diseases progression. Ideally, this can be achieved through experimental results that depict valuable methods with better performance when compared with other studies. In the same context, many strategies were developed to achieve such objectives by employing novel statistical models on large-scale datasets [1–6]. Such an observation has prompted the requirement of effective patient management protocols, especially for those admitted into intensive care unit. However, the same protocols are not fully applicable to Non–Intensive Care Unit (Non-ICU) inpatients, and this has inculcated poor inpatient management practices regarding the number of treatments, the number of lab test conducted, discharge, insignificant changes or improvements at the time of discharge, and high rates of re-admissions. Nonetheless, such a claim has not been proven and the influence on these factors on re-admission among diabetes. As such, this study hypothesized that time spent in hospital, number of lab procedures, number of medications, and number of diagnoses have an association with re-admission rates and are proxies of in-hospital management practices that affect patient health outcomes. However, detection of Hemoglobin A1c (HbA1c) marker, administration of insulin treatment, diabetes treatment instances, and noted changes are factors that can moderate the admission and are treated as partial management factors in the study. Some of the re-admission is avoidable although this requires evidence-based treatments. According to [7] in a retrospective cohort study evaluated the basic diagnoses and 30-day re-admission patterns among Academic Tertiary Medical Center patients’ and established within 30-days re-admissions are avoidable. In specific, the study established that 8.0% of the 22.3% of the within 30 days re-admissions are potentially avoidable. As a subtext to the conclusion, the authors asserted that these re-admission cases were related in direct or indirect consequences due to the pre-conditions related to the primary diagnosis. For instance, research demonstrated that patients admitted for heart failure and other related diseases are more likely to be readmitted for acute heart failure.However, the re-occurrence of the heart condition is dependent on the treatment administered, observed health outcome at discharge, and other pre-existing health conditions.

## Research contribution

Under the circumstances, it is essential for healthcare stakeholders to pursue re-admission reduction strategies, especially with a specific focus on the potentially avoidable re-admissions. The authors in [8] highlighted the role that financial penalties imposed on health institutions with higher re-admission rates in reducing the re-admission incidences. Furthermore, the article assessed and concluded that extensive assessment of patient needs, reconciling medication, educating the patients, planning timely outpatient appointments, and ensuring follow-up through calls and messages are among the best emerging practices for reducing re-admission rates. However, implementing these strategies requires significant funding although the long-term impacts outweigh any financial demands. Hence, it suffices to deduce that re-admissions in a health facility are a priority area for improved health facilities and reducing healthcare cost. Regardless of the far-reaching interest in hospital re-admissions, little research has explored re-admission among diabetes patients. A reduction of diabetic patient re-admission can reduce health cost while improving health outcomes at the same time. More importantly, some studies have identified socioeconomic status, ethnicity, disease burden, public coverage, and history of hospitalization as key re-admission risk factors. Besides these factors and principal admission conditions, re-admission can be a factor of health management practices. This study provides information on the managerial causes of re-admission using six machine learning models. Additionally, most studies employ regression data mining technique and as such this study provides a framework for implementing other machine learning techniques in exploring the causative agents of re-admission rates among diabetes patients. The primary importance of the algorithm is to help hospitals identify multiple strategies that work effectively for re-admission of a given health condition. In specific, implementation of multiple strategies will focus on improved communication, the safety of the medication, advancements in care planning, and enhanced training on the management of medical conditions that often lead to re-admissions. Each of these sub-domains involves decision making and given the size and nature of healthcare information, data mining and deep learning techniques may prove critical in reducing the re-admission rates.

## Methodology

**Fig. 1:**
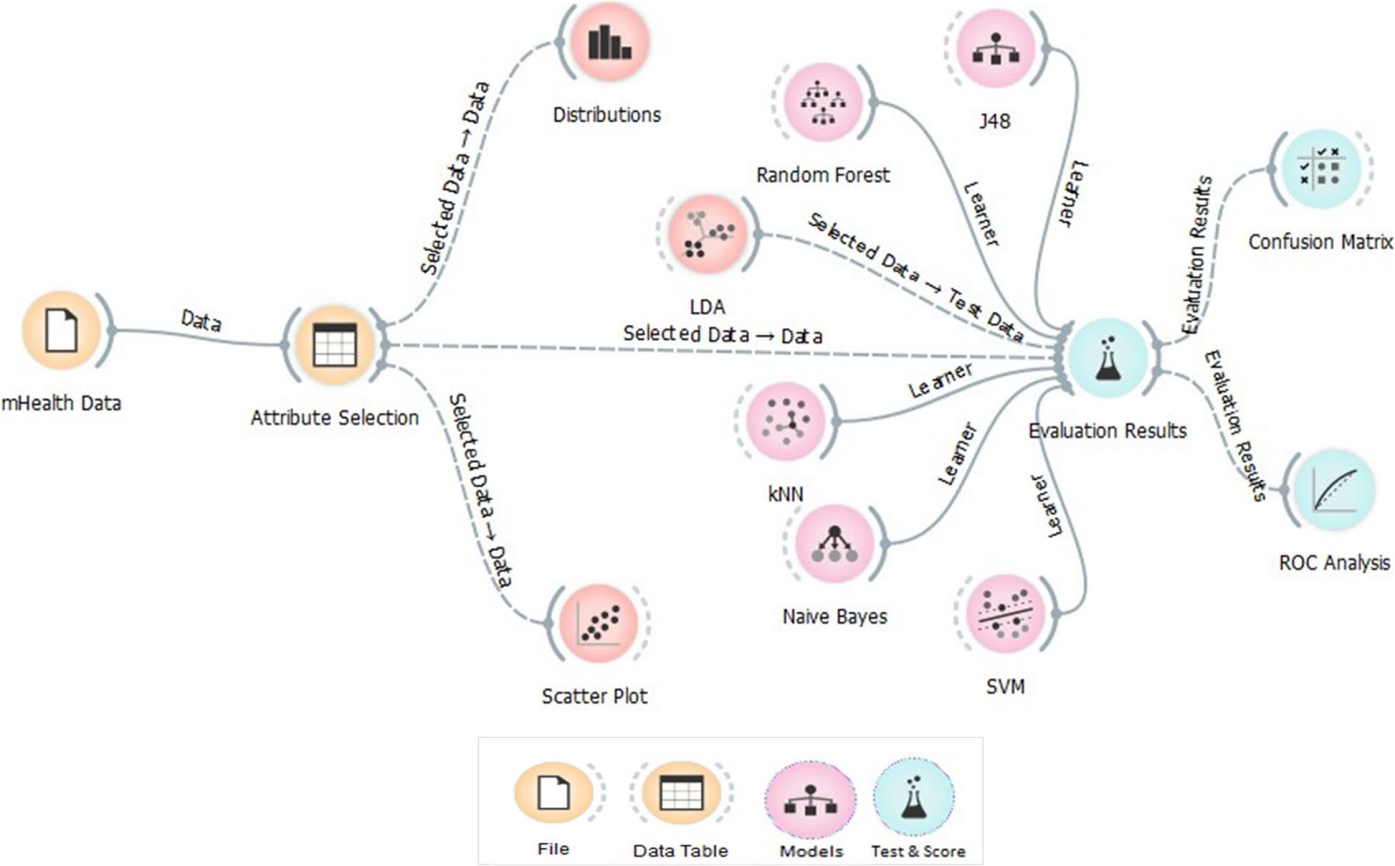
The Machine Learning Process Diagram

Figure 1 illustrates the high-level machine learning process diagram used in the paper. The study explored the probable predictors of diabetes hospital re-admission among the hospitals using machine learning techniques along with other exploratory methods. The dataset consists of 55 attributes and only 18 were used as per the scope of the study. The performance of the models is evaluated using the conventional confusion matrix and ROC efficiency analysis. The final re-admission model is based on the best performing model as per the true positive rates, sensitivity and specificity.

### Linear discriminant analysis

LDA algorithm is a variant of Fisher’s linear discriminant and it classifies data to vector format based linear combination of attributes based on a target factor or class variable. The algorithm has a close technical resemblance to Analysis of Variance (ANOVA) and regression as it explains the influences of predictors using linear combinations [5]. There are two approaches to LDA. The techniques assume that the data conforms to Gaussian distribution and as such, assumes that each attribute has a bell-shape curve when visualized and it also assumes that each variable has the same variance, and that data points of each attribute vary around the average by the same amount. That is, the algorithm requires the data and its attributes to be normally distributed and of constant variance or standard variation. As a result, the algorithm estimates the mean and the variance of the data for each of the class that it creates using the conventional statistical techniques.

1 $$ \mu=\frac{1}{nk}\sum(x)   $$

Where *μ* is the mean of each input attribute (*x*) for each class (*k*) and *n* is the total number of observations in the dataset. The variance associated with the classes is also computed using the following conventional method.

2 $$ \sigma^{2}=\ \frac{1}{n-k}\ \sum{(x-\mu})^{2}   $$

In Eq. 2, sigma squared is the variance across all instance serving as input in the model, *k* is the number of classes, and *n* is the number of observations or instance in the dataset. *μ* is the mean and is computed using Eq. 1.

Besides the assumptions, the algorithm makes prediction using a probabilistic approach that can be summarized in two steps. Firstly, LDA classifies predictors and assigns them to a class based on the value of the posterior probability denoted as

3 $$ \pi\ \left(y=\complement_{i}\middle| x\ \right)   $$

The objective is to minimize the total probability of mis-classifying the features, and this approach relies on Bayes’ rule and the Gaussian distribution assumption for class means where:

4 $$ \pi\ \left(x\middle| y\ =\ \complement_{i}\right)   $$

Secondly, LDA finds a linear combination of the predictors that return the optimum predictor value, and this study uses the latter. LDA algorithm can be implemented in five basic steps. First, in performing LDA classification, the d-dimensional mean vectors are computed for the classes identified in the dataset using the mean approach (Eq. 1). The variance and the normality assumption must be checked before proceeding. Second, both within and between-class scatters are computed and returned as a matrix. The within-class scatter or distances are computed based on Eq. 5.

5 $$ S_{within\ =\ }\sum_{i=1}^{c}S_{i}   $$

and

6 $$ S_{i}\ =\ \sum_{x\in D i}^{n}{\left(x\ -\ \mu_{i}\ \right)(x\ -\ \mu_{i})^{T}}   $$

where *i* is the scatter for every class identified in the dataset and *μ* is the mean of the classes computed using Eq. 1.

The Between-class scatter is calculated using Eq. 7.

7 $$ S_{between}\ =\ \sum_{i-1}^{c}{N_{i}\left(\mu_{i}\ -\ \mu\ \right)(\mu_{i}\ -\ \mu)^{T}}   $$

In Eq. 7, *S* is general mean value while *μ* and *N* refers to the sample mean and sizes of identified classes respectively. The third step involves solving Eigenvectors associated with the product of the within-class and out-class matrices. The fourth step involves sorting the linear discriminant to identify the new feature subspace. The selection and sorting using decreasing magnitudes of Eigenvalues. The last step involves the transformation of the samples or observations onto the new linear discriminant sub-spaces. The pseudo-code for LDA is presented in Algortihm 1.


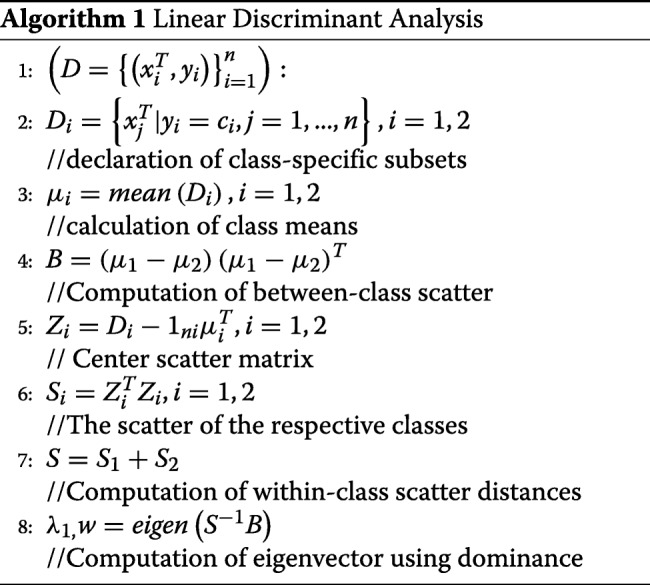


For the classes *i*, the algorithm divides the data into *D*_1_ and *D*_2_ then calculates the within and between the class distances, and the best linear discriminant is a vector obtained from the product of transpose of within-class and between-class scatter matrices.

#### Random forest

Random forest is a variant of decision degree growing technique and it is different from the other classifiers, because it supports random growth branches within the selected subspace. The random forest model predicts the outcome based on a set of random base regression trees. The algorithm selects a node at each random base regression and split it to grow the other branches. It is important to note that Random Forest is an ensemble algorithm because it combines different trees. Ideally, ensemble algorithms combine one or more classifiers with the different types. Random forest can be thought of a bootstrapping approach for improving the results obtained from the decision tree. The algorithm works in the following order. First, it selects a bootstrap sample *S*^(*i*)^from the sample space and the argument denoting the bootstrap sample refers to the *i*^*th*^ bootstrap. The algorithm learns a conventional decision tree although through implementation of a modified decision tree algorithm. The modification is specific and is systematically implemented as the tree grows. That is, at each node of decision tree, instead of implementing an iteration for all possible feature split, RF randomly selects a subset of features such that *f*⊆*F* and then splits the features in the subset (*f*). The splitting is based on the best feature in the subset and during implementation, the algorithm chooses the subset that it is much smaller than the set of all features. Small size of subset reduces the burden to decide on the number of features to split since datasets with large size subsets tend to increase the computational complexity. Hence, the narrowing of the attributes to be learned improves the learning speed of the algorithm.


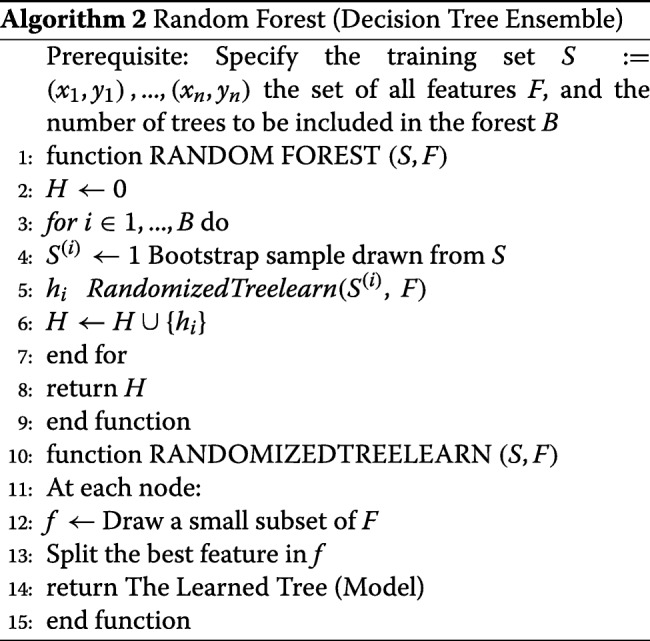


The algorithm uses bagging to implement the ensemble decision tree, and it is prudent to note that bagging reduces the variance of the decision tree algorithm.

### Support vector machine

Support Vector Machine is a group of supervised learning techniques that classify data based on regression analysis. One of the variables in the training sample should be categorical so that the learning process assigns new categorical value as part of the predictive outcome. As such, SVM is a non-likelihood binary classifier leveraging the linear properties. Besides classification and regression, SVM detects outliers and is versatile when applied to dimensionality high [1]. Ideally, a training vector variable, that has at least two categories, is defined as follows:

8 $$ x_{i}\in\mathbb{R}^{p},i=1,...,n   $$

where *x*_*i*_ represents the training observation and *R*^*p*^ indicates the real-valued p-dimensional feature space and predictor vector space. A pseudo-code for a simple SVM algorithm is illustrated:


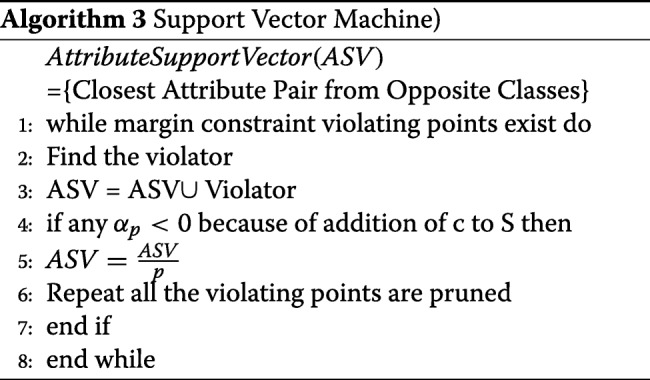


The algorithm searches for candidate support vectors denoted as *S* and it assumes that *SV* occupies as a space where the parameters of the linear features of the hyper-plane are stored.

### k-nearest neighbor

kNN classifies data using the same distance measurement techniques as LDA and other regression-based algorithms. In classification application, the algorithm produces class members while in regression application it returns the value of a feature or a predictor [9]. The technique can identify the most significant predictor and as such was given preference in the analysis. Nonetheless, the algorithm requires high memory and is sensitive to non-contributed features despite being considered insensitive to outliers and versatile among many other qualifying features. The algorithm creates classes or clusters based on the mean distance between data-points. The mean distance is calculated using the following equation.

9 $$ \mathrm{\Psi}(x)=\frac{1}{k}. {\sum}{\left(x_{i},y_{i}\right)\in kN N(x,L,K)} y_{i}   $$

In Eq. 9, *kNN*(*x*,*L*,*K*), *k* denotes the *K* nearest neighbors of the input attribute (*x*) in the learning set space (*i*). The classification and prediction application of the algorithm depends on the dominant *k* class and the predictive equation is as the following:

10 $$ \mathrm{\Psi}(x)={argmax}{c\in y}.{\sum}{\left(x_{i},y_{i}\right)\in N\left(x,L,K\right)} y_{i}   $$

It is imperative to note that output class consists of members from the target attribute and the distance used in assigning the attributes to classes is based on Euclidean distance. The implementation of the algorithm consists of six steps. The first step involves the computation of Euclidean distance. In the second step, the computed n distances are arranged in a non-decreasing order, and in the third step, a positive integer *k* is drawn from the sorted Euclidean distances. In the fourth step, k-points corresponding to the k-distances are established and assigned based on proximity to the center of the class. Finally, for *k* >0 and for (number of points in the *i*, an attribute *x* is assigned to that class if *k*_*i*_>*k*_*j*_*for all* *i*≠*j* is true. Algorithm 4 shows the kNN steps process:


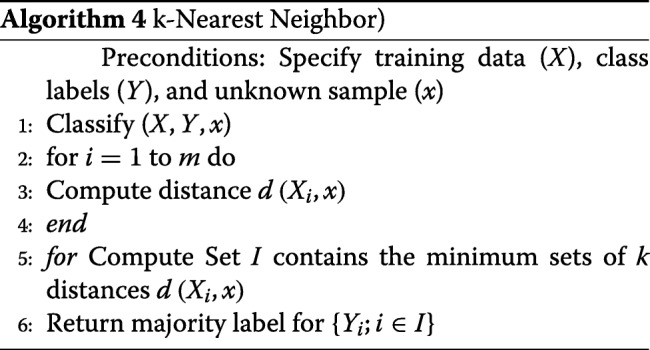


### Naïve Bayes

Even though Naïve Bayes is one of the supervised learning techniques, it is probabilistic in nature so that the classification is based on Naïve Bayes’ rules of probability, especially those of association. Conditional probability is the construct of Naïve Bayes classifier [9–16]. The algorithm assigns instance probabilities to the predictors parsed in a vector format representing each probable outcome. Naïve Bayes classifier is the posterior probability that the dividend of the product of prior with likelihood and evidence returns. The construction of the model from the output of the analysis is quite complex although the probabilistic computation from the generated classes is straightforward [17–22]. The Bayes Theorem upon which the Naïve Bayes classifier is based can be written as follows:

11 $$ P\left(\mu|\nu\right)=\frac{P(\nu|\mu)P(\mu)}{P(\nu)}   $$

Where *μ* and *v* are events or instances in an experiment and *P*(*μ*) and *P*(*ν*) are the probability of their occurrence. The conditional probability of an event *μ* occurring after *v* is the basis of Naïve Bayes classifier. The classifier uses maximum likelihood hypothesis to assign data points to classes. The algorithm assumes that each feature is independent and makes equal contribution to the outcome or all features belonging to the same class have the same influence on that class. In Eq. 11, the algorithm computes the probability of event *μ* provided that *v* already occurred, and as such *v* is the evidence and the probability *P*(*μ*) is regarded as the priori probability. That is, it refers to probability obtained before seeing the evidence while the conditional probability *P*(*μ*|*ν*) is priori probability of *v* since it is a probability computed with evidence.

### J48

J48 is one of the decision tree growing algorithm. However, J48 is the reincarnation of the C4.5 algorithm, which is an extension of the ID3 algorithm [23]. As such, J48 is a hierarchical tree learning technique and it has several mandatory parameters including the confidence value and the minimum learning instance, which are translated to branches and nodes in the final decision tree [23–29].

## Data assembly and pre-processing

The study used diabetes data that was collected across 130 hospitals in the US in the years between 1999–2008 [30]. The dataset includes data systematically composed from contributing electronic health records’ providers that contained encounter data such as inpatient, outpatient and emergency, demographics, provider specialty, diagnosis, in-hospital procedures, in-hospital mortality, laboratory and pharmacy data. The complete list of the features and description is provided in Table S1 (Additional file 1). The data has 55 attributes, about 100,000 observations, and has missing values. However, the study used a sample based on the treatment of diabetes. In specific, of the 100,000 cases, 78,363 meet the inclusion criteria since they received medication for diabetes. Consequently, the study explored re-admission incidences among patients who had received treatment. The amount of missing information, the type of the data (categorical or numeric) that guided the data cleaning process, re-admission, Insulin prescription, HbA1c test results, and observed changes were retained as the major out-come associated with time spent in the hospital, the number of diagnoses, lab procedures, procedures, and medications [31, 32]. Of the 55, only 18 variables were selected as per the scope for analysis and even about 8 of the selected served as proxy controls. The study was split into 70% training and 30% validation subsets.

## K-fold validation

**Fig. 2:**
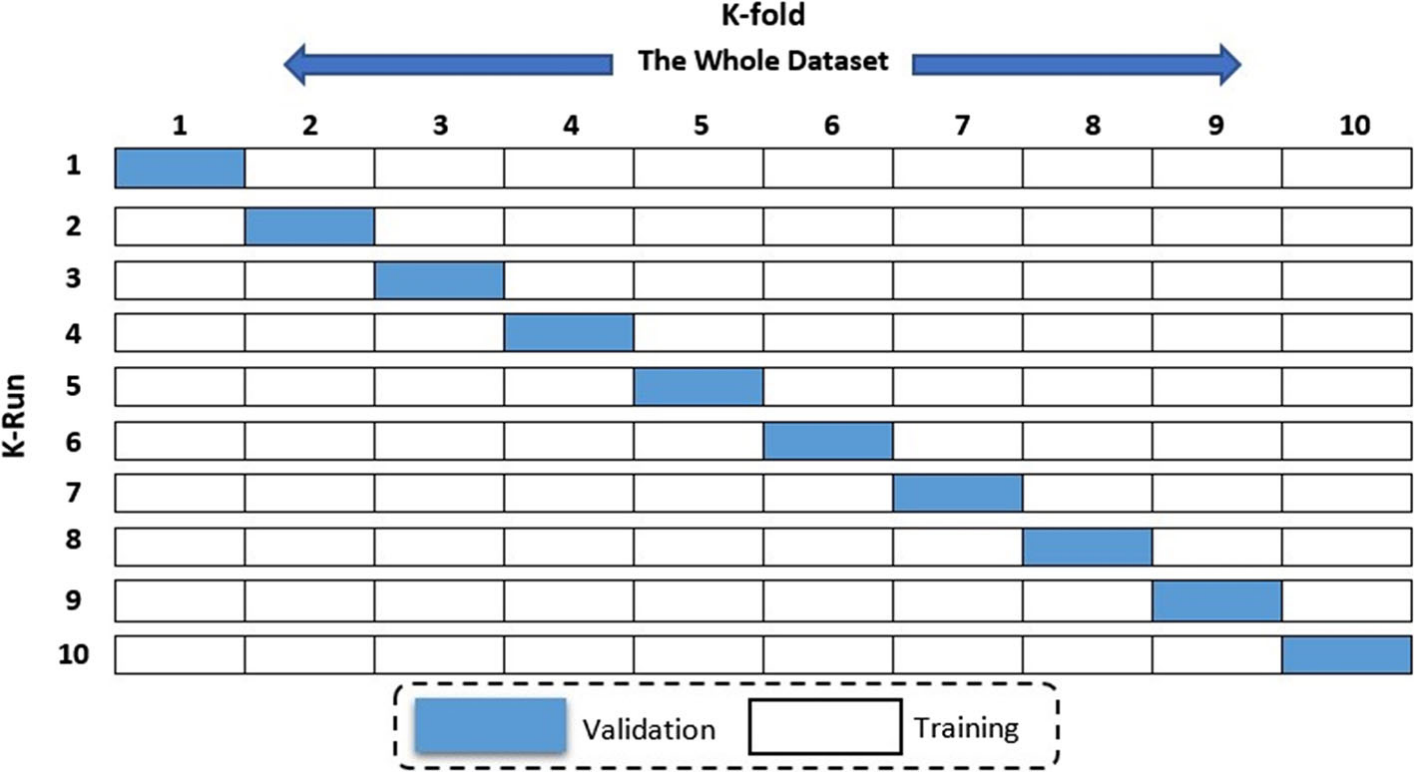
Cross-Validation Scheme for both training validation subsets

To improve the overall accuracy and validate a model, we relied on the 10-fold cross validation method applied for estimating accuracy. The training dataset is split into k-subsets and the subset held out while the model is fully trained on remaining subsets. Figure 2 illustrates the validation method. The K-fold Cross-validation method utilizes the defined training feature set and randomly splits it into k equal subsets. The model is trained k times. During each iteration, 1 subset is excluded for use as validation. This technique reduces over-fitting issues, which occurs when a model trains the data too closely to a set of data, which can result in failure to predict future information reliably [2, 12, 33].

## Discussion

### Exploratory analysis

Of the 47.7% diabetic patients who were readmitted, 11.6% stayed in the hospital for less than 30 days while 36.1% stayed for more than 30 days. A majority (52.3%) of those who stayed for more than 30 days did not receive any medical procedures during the first visit. In general, diabetic patients who received a fewer number of lab procedures, treatment procedures, medications, and diagnoses are more likely to be readmitted than their counterparts. Furthermore, the more frequent a patient is admitted as an in-patient the less likely the probability of re-admission. Our study indicated that, women (53.3%) and Caucasian (74.6%) diabetic patients are more vulnerable to re-admission than male and the other races. Besides several lab procedures, medications, and diagnoses, insulin administration and HbA1c results exacerbate the re-admission rates among diabetic patients.

### Scatterplots

**Fig. 3:**
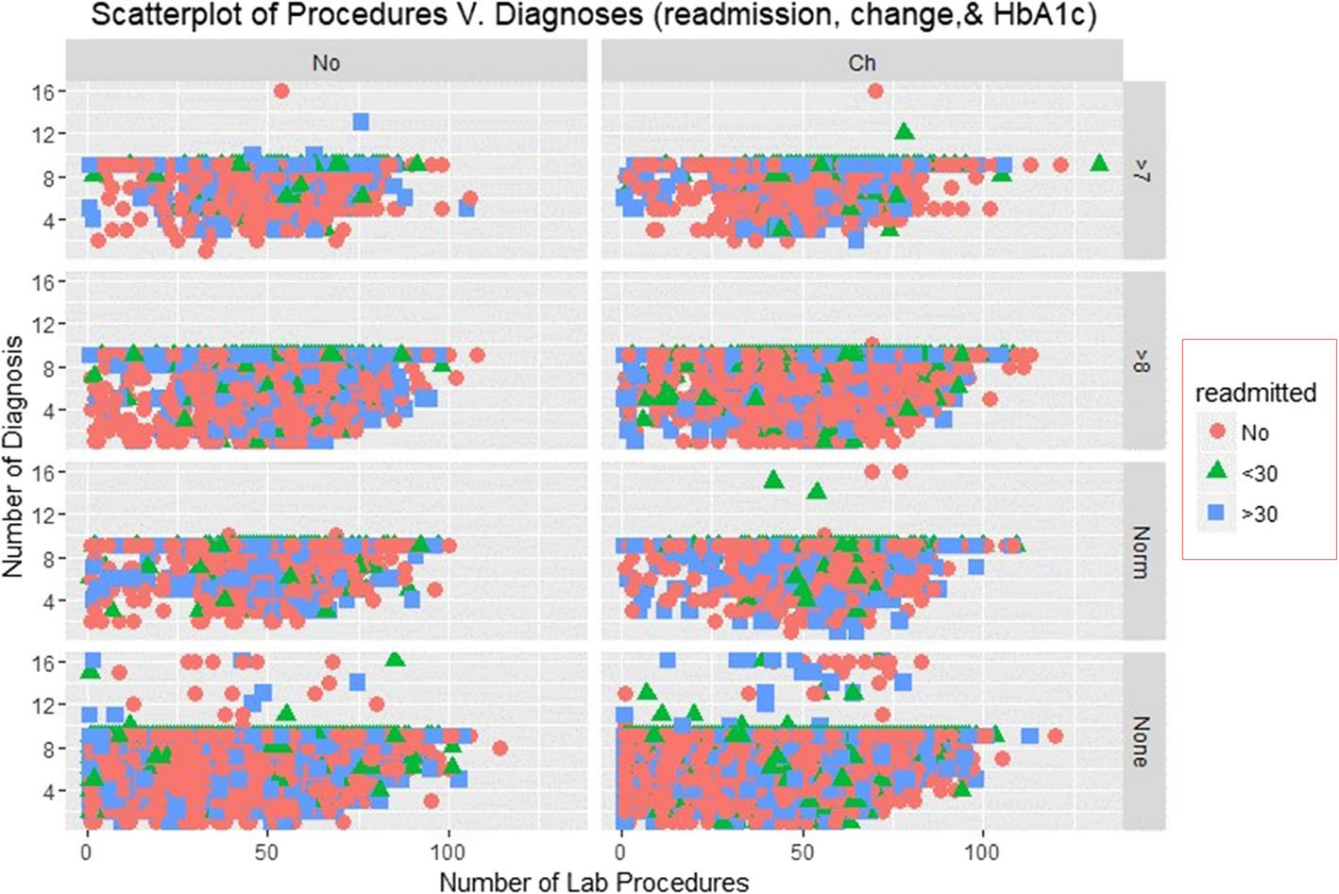
Scatterplot of Medications and Diagnoses

**Fig. 4:**
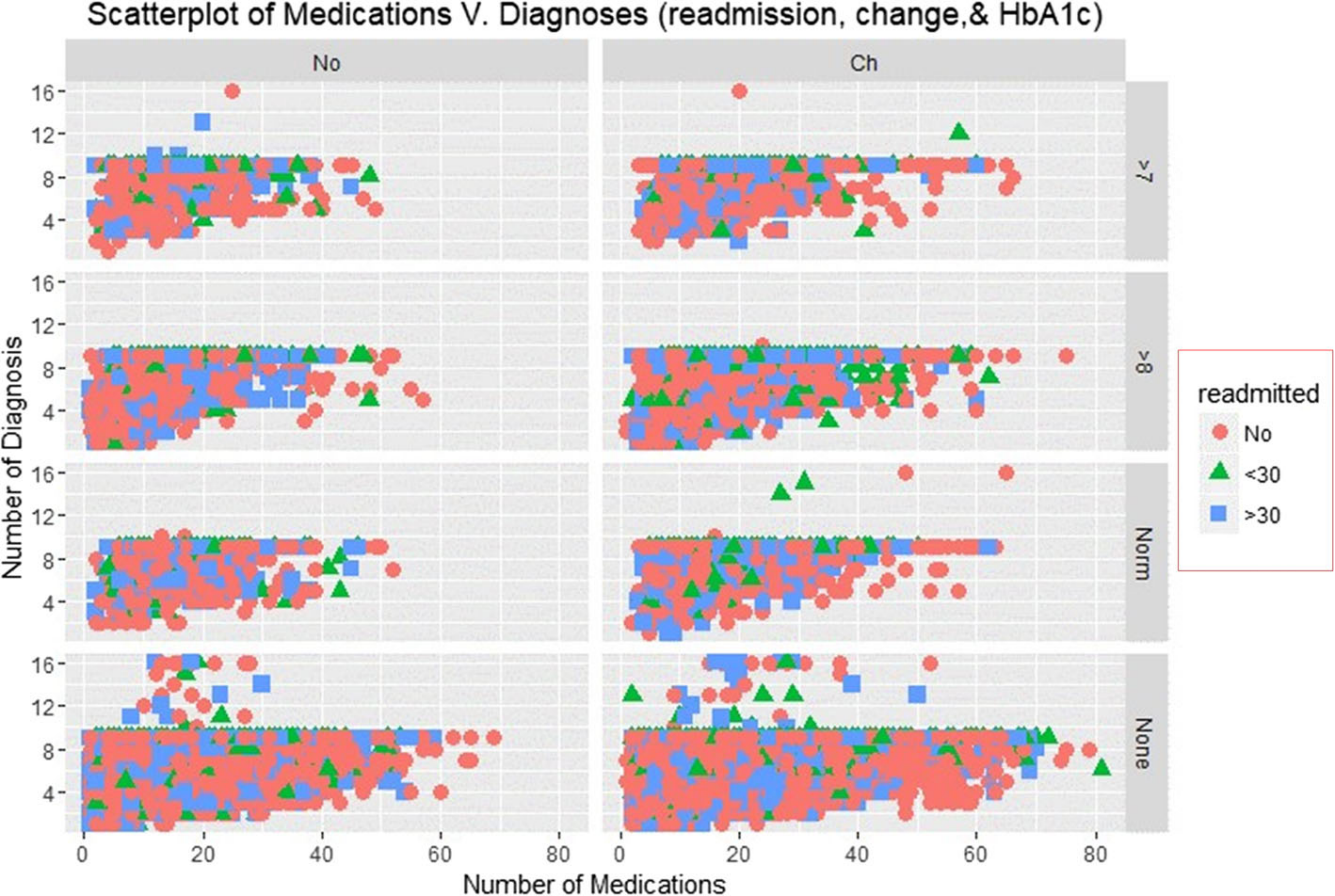
Scatterplot of Medications and Diagnoses

The Scatterplots of re-admission incidences with an overlay of HbA1c measurements and change recorded at the time of discharge are shown in Figs. 3 and 4.

Figure 3 illustrates the Scatterplot of the number of diagnoses and lab procedures that patient received for re-admission rates. The figures have 8 panels displaying scatters of diagnoses and lab procedures for different instances of HbA1c results and change. The plot shows that patients who had negative HbA1c tests results received several diagnosis and very few were readmitted. Those who received less than 10 diagnoses and less than 70 procedures were more likely to be readmitted. None of the patients received more than diagnosis and a majority were admitted for more than 30 days.

Figure 4 depicts a scatter plot of a number of diagnoses and lab procedures. The re-admission rates are quite different between a group of patients who noted change at discharge than those who did not. Those who failed to note significant improvement at discharge received more than 50 medications and less than 10 diagnoses. However, re-admission is higher among those who noted improvement at discharge.

### Density distributions

**Fig. 5:**
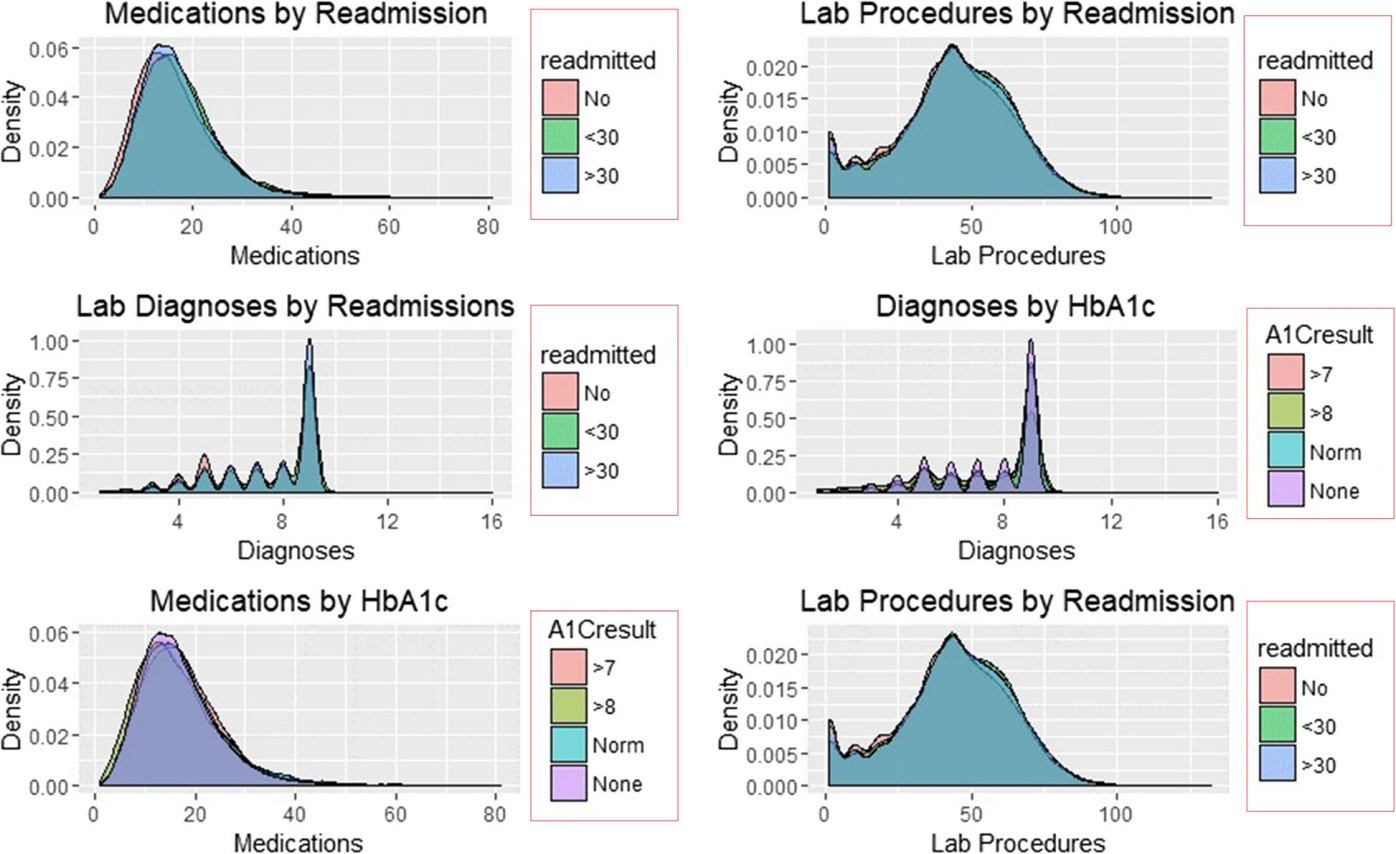
Density Plots of Predictors by re-admission and HbA1c

**Fig. 6:**
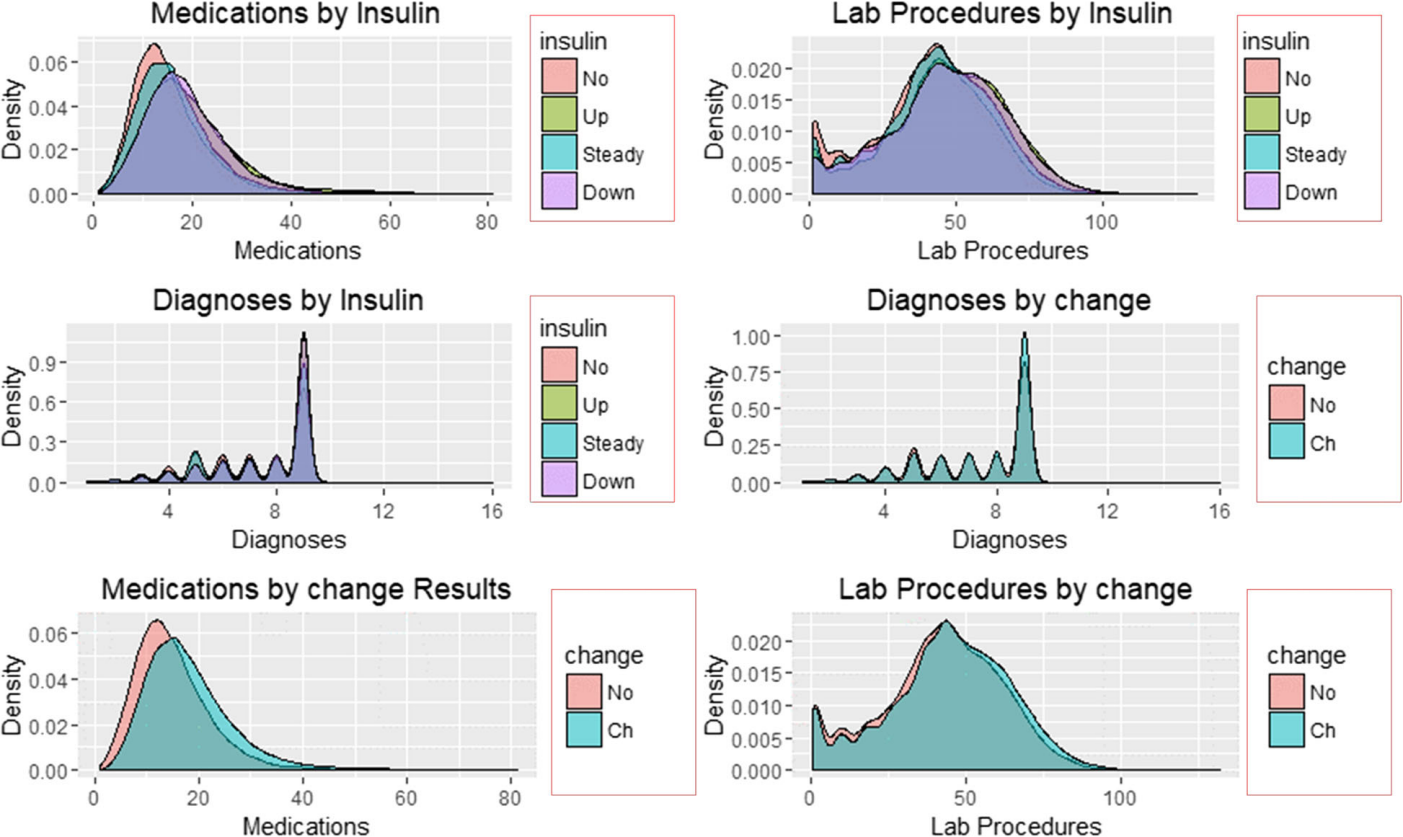
Density Plots of Predictors by Insulin and change

The distribution of re-admission and subsequent patterns associated with reported change and results of HbA1c are shown in Figs. 5 and 6.

Figures 5 and 6 illustrate the density distribution of number of medications, lab procedures, and diagnoses grouped by re-admission, HbA1c results, insulin administration change at discharge. Notably, the distribution density of the number of lab procedures, medications, and diagnoses are the same for grouping categories. Figure 6 shows significant differences in the number of medications and lab procedures. For instance, the average number of medications differs between ’No’, ’Up’, ’Steady’, and ’Down’ insulin categories. A similar difference in mean of the number of medications is observed in the change distribution curve with those recording change at discharge receiving more medications than their counterparts.

### Smooth linear fits

**Fig. 7:**
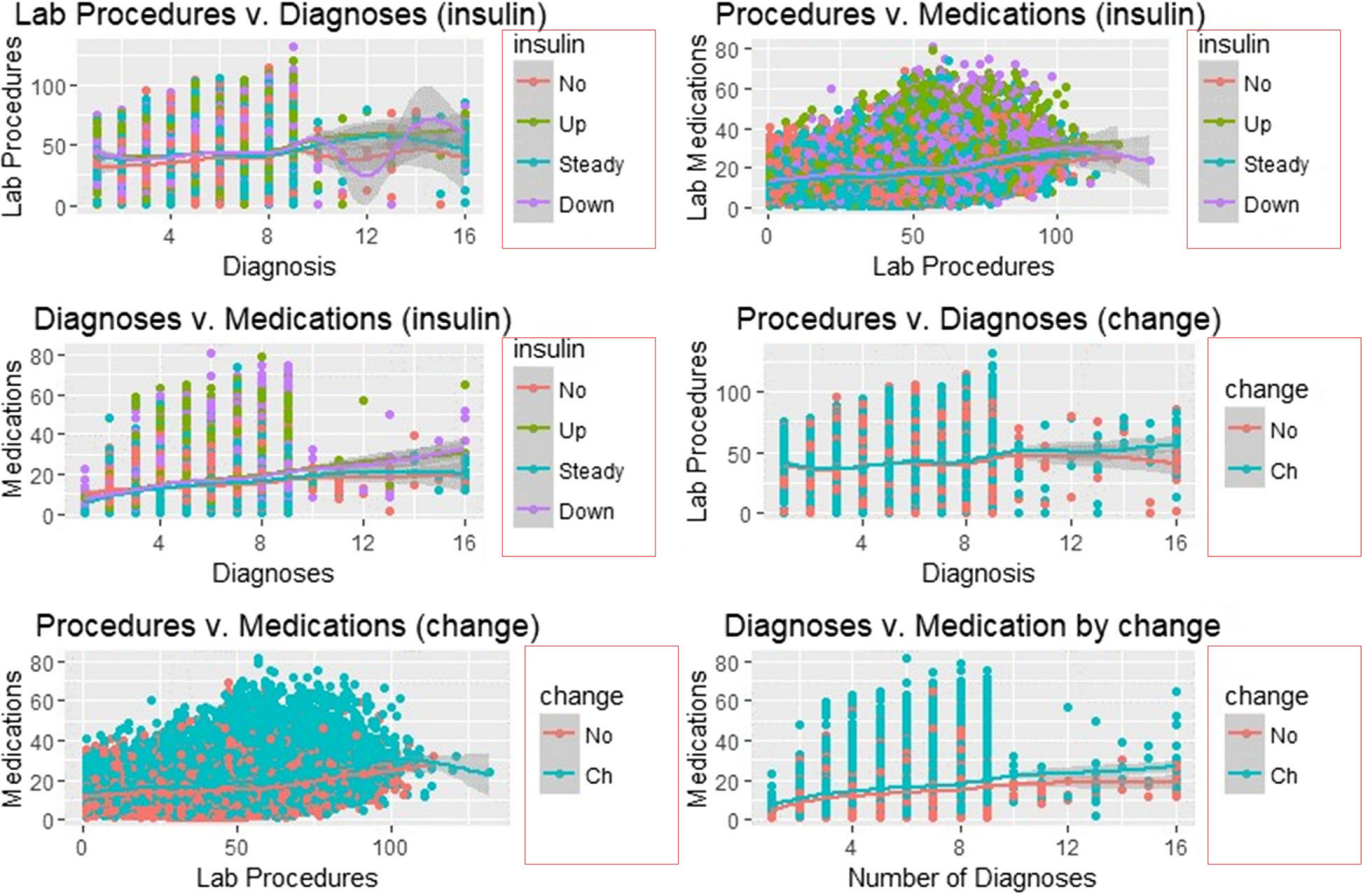
Smooth Linear Fits with Insulin and Change as Facets

**Fig. 8:**
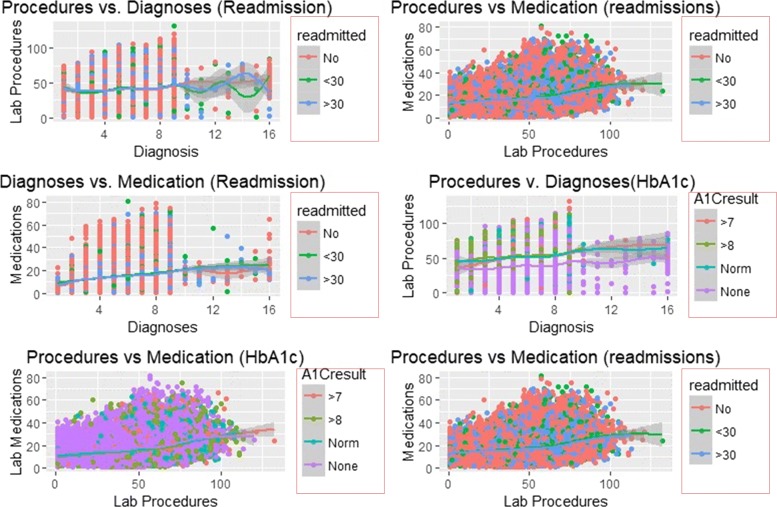
Smooth Linear Fits with re-admission and HbA1c as Facets

Figures 7 and 8 illustrate the smooth line fits associated with Scatterplots. The smoothen fits include a 95% confidence interval and demonstrates the likely performance of linear regression models in forecasting re-admission.

Figures 7 and 8 depict smooth linear fits of the Scatterplots and density plots in Figs. 3, 4, 5, and 6. The figures illustrate that the number of lab procedures has linear relationships with the number of diagnoses although the data is likely to be heteroskedastic. The number of diagnoses and medications also have the same relationship and plot patterns. For medication versus procedures, the relationship is linear and change in diabetes status increases with medications and lab procedures. As for re-admission, incidents of more than 30 days re-admission reduced with increasing number of diagnoses, lab procedures, and medications. Similarly, the probability of detecting HbA1c increases with increasing number of diagnoses and lab procedures.

### Model evaluation

The performance of the models in predicting re-admission incidence was based on the confusion matrix and in specific the percentage of the correctly predicted read-mission categories.

Table 1 depicts that Naïve Bayes correctly classified the re-admission rates less than 30 days and none re-admission incidences. SVM accurately classified 48.3% of the re-admission incidence exceeding 30 days. The objective is to obtain the performing model.

**Table 1:** True Positive Rate Comparison Table

Model	<30 days	>30 days	No
Random Forest	21.0%	42.8%	60.5%
kNN	17.8%	40.3%	59.6%
Naïve Bayes	23.6%	46.6%	61.2%
SVM	12.2%	48.3%	55.9%
J48	17.3%	40.4%	60.3%

### Individual model performance

The LDA model yields two linear discriminants LD1 and LD2 with proportion trace of 0.9646 and 0.0354 respectively. Hence, the first LD explains more than 96.46% of the between-group variance while the second account for 3.54% of the between-group variance.

12 $$  {}\begin{aligned} LD_{1}=003*\text{ Lab Procedures }-0.102*{ Procedures }\\ + 0.08*\text{ Medications} +0.18 *\text{ Emergency }+0.67\ \text{ Inpatient }\\ + 0.17 \text{Diagnoses} \end{aligned}  $$

**Fig. 9:**
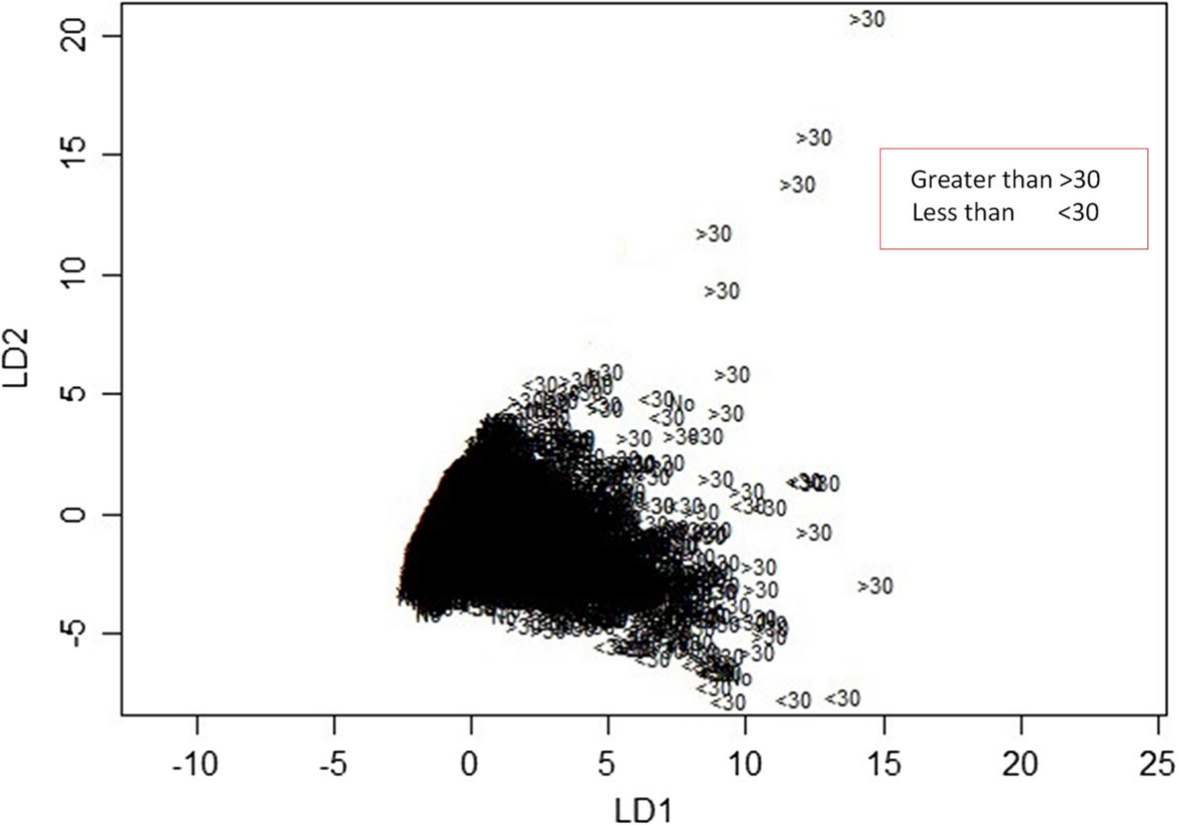
Plot of two linear discriminants obtained from LDA learner

Figure 9 illustrates the plot of LD1 versus LD2. Equation 12 depicts the profile of diabetic patients.

The predictors were significantly correlated at 5% level and they influenced re-admission based on the frequency of each. The kNN model used all the 16 predictors to learn the data and selected three as significant predictors. In specific, the kNN model proposes that high re-admission for diabetes treatment is caused by a fewer number of lab procedures, diagnoses, and medications. However, the rates are higher among patients who tested positive for HbA1c and did not fail to receive insulin treatment (Fig. 3).

SVM classified the readmitted diabetic patients into three classes using a polynomial of degree 3 suggesting that diabetes re-admission cases do not have a linear relationship with the predictors. As an inference, the polynomial relationship illustrated by the kernel and degree of the SVM indicates higher re-admission rates among patients discharged without any significant changes (Fig. 4). Naïve Bayes classifier yields two classes using the Laplace approach. The classification from the model depicts a reduced likelihood of re-admission in cases where the patients undergo a series of laboratory tests, rigorous diagnosis, proper medication, and discharge after confirmation of improvement. The density distributions in Figs. 5 and 6 compliments the findings of the model. In specific, the distributions of the number of medications and lab procedures show a noticeable difference in the distribution when considering insulin administration as part of treatment. Regarding aggregation of the distribution of the number of medications and lab procedures by status at discharge (change), the distribution curves suggest that patients are more likely to feel better at time of discharge provided that the lab services and medications are of superior quality. It is important to reiterate that Naves Bayes’ model has true positive and false negative rates showing that it had 13.78% accuracy and 13.78% sensitivity. Finally, random forest classified diabetic patients using linear approaches with re-admission as the control. Figures 7 and 8 demonstrate that the smoothen linear of the paired predictors shows that re-admissions taking more than 30 days is reduced by increasing the number of medical diagnoses. Further, the HbA1c results increase with increasing number of diagnoses. However, it is important to note that the association between the number of lab procedures and medications tends to be non-linear while that between the number of diagnoses and medication is linear regardless of the grouping variable. The J48 based tree shown in Fig. 9 does not consider the linear relationships and omits diabetic patients who were never re-admitted. The resultant tree included a number of inpatient treatment days, number of emergencies, number of medications, lab procedures, and diagnoses in the model. The model suggests that diabetic patients admitted as in-patients tend not to be re-admitted. Similarly, the tree demonstrates that several diagnoses improve health outcomes and reduce re-admission.

### Best fit model

The best fitting model is based on the performance measures summarized in Table 2. The key decision relies on the efficiency of the model in predicting the re-admission rates and the area under the curve (AUC) and precision/recall curve are the best measures for such a task.

**Table 2:** Comparison of model efficiency and sensitivity

Model	AUC	CA	F1	Precision	Recall
kNN	0.575	0.499	0.489	0.482	0.499
J48	0.578	0.490	0.487	0.485	0.490
SVM	0.547	0.475	0.421	0.483	0.475
Random Forest	0.602	0.529	0.509	0.499	0.529
Naïve Bayes	0.640	0.566	0.524	0.519	0.566

Table 2 illustrates that Naïve Bayes is the most sensitive and efficient model for learning, classifying and predicting re-admission rates using mHealth data. It has an efficiency of 64% and a sensitivity of 52.4%. The ROC curves associated with the predictions of re-admission that exceeded 30 days are displayed in the figures below.

**Fig. 10:**
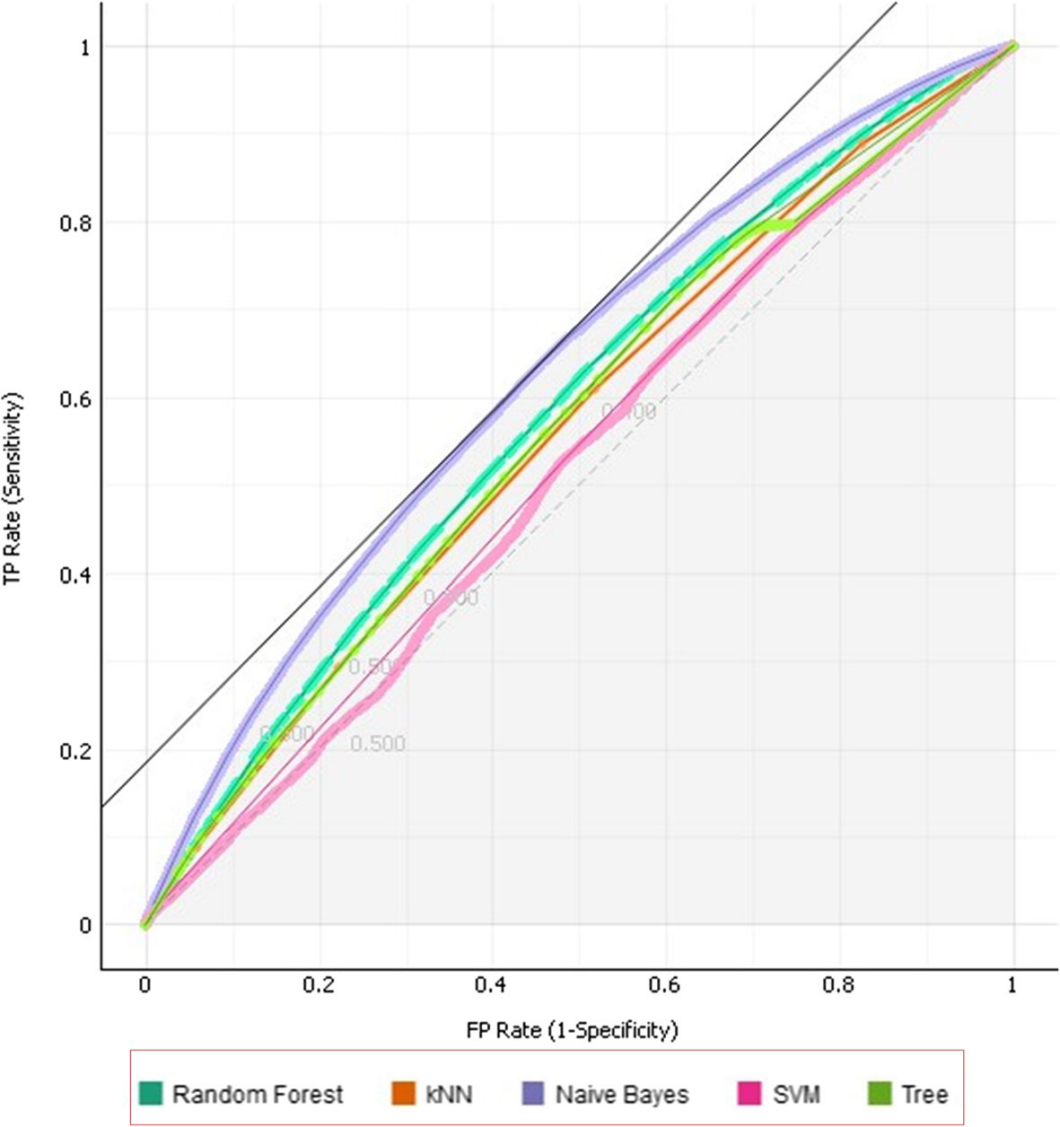
ROC curves illustrating the Areas Under Curve for the models

The larger the area covered the more efficient the model is, and this principle Fig. 10 depicts that Naïve Bayes is the most efficient.

### Naïve bayes analysis

**Fig. 11:**
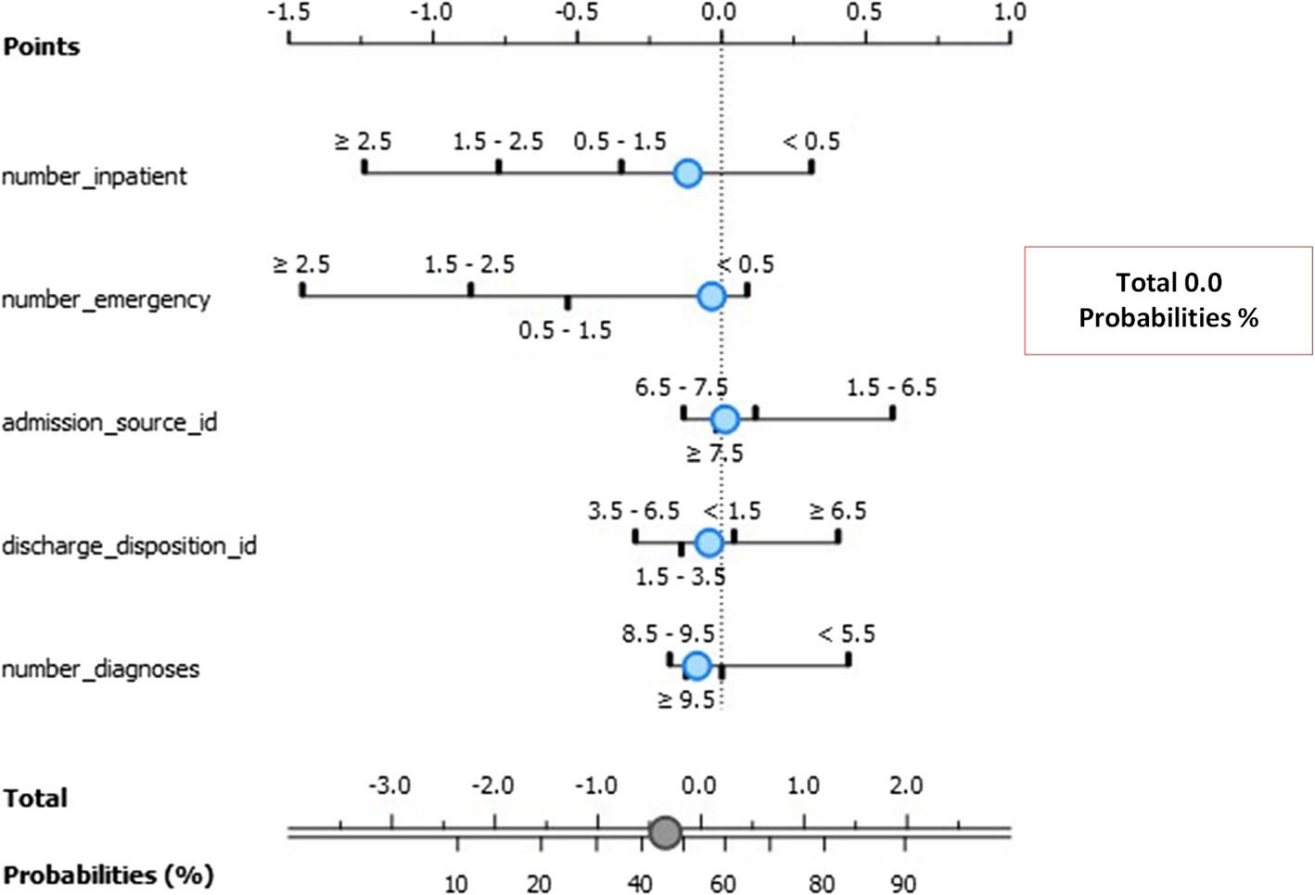
Nomogram visualization of Naïve Bayes classifier on target class 0

The model focused on the top 5 best factors (exposures) that contributed to re-admission for less and more than 30 days. The association between the exposures and outcome (re-admission instances) are given as log odds ratio in the nomograms illustrated in Fig. 11. The three classes model are Class 0 (No re-admission), Class 1 (re-admission for less than 30 days), and Class 3 (re-admission for more than 30 days).

Figure 11 depicts the exposure factors with absolute importance on Class 0 including number of emergencies, number of patients, discharge disposition ID, admission source ID, and number of diagnoses. The log odds ratios illustrate the association between these exposure factors. The conditional probability for re-admission after discharge based on these exposure factors is 0.5.

**Fig. 12:**
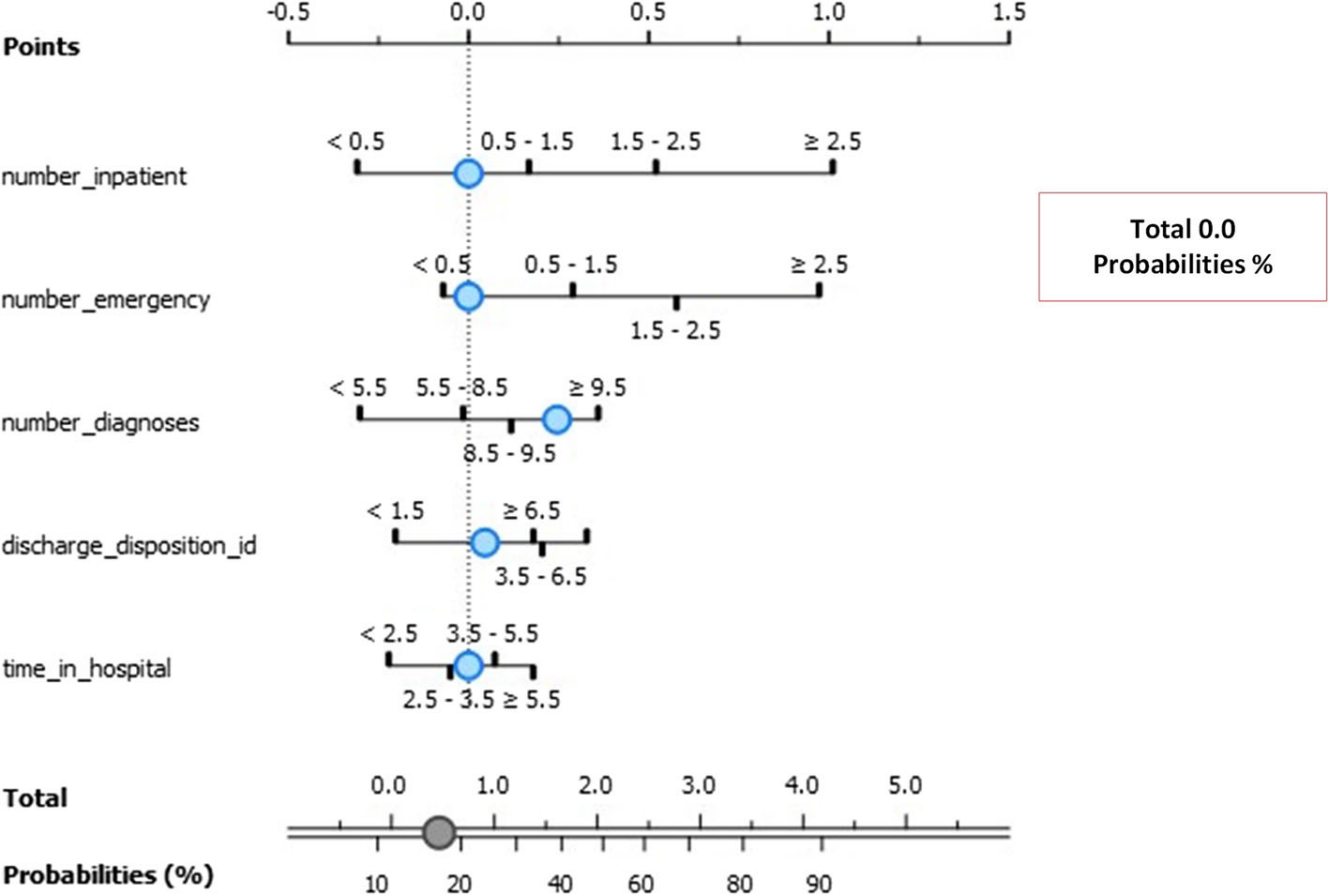
Nomogram visualization of Naïve Bayes classifier on target class 1

Figure 12 depicts the exposure factors with absolute importance on Class 1 including the number of emergencies, the number of patients, discharge disposition ID, time in hospital ID, and number of diagnoses. The log odds ratios display the association between these exposure factors to lack of re-admission after discharge. The conditional probability for re-admission after discharge based on these exposure factors is 14%. In specific, there is a 48% chance of re-admission for patients with a number of diagnoses between 8.5 and 9.5, and a 52% chance for those with diagnoses between 5.5 and 8.5. Similarly, those spending between 2.5 to 3.5 days in the hospital is more likely to be readmitted (59%) for less than 30 days than their counterparts with 41% chance of re-admission. Finally, those with fewer emergency admission history stand higher chances of re-admission (80%) than those with sufficient emergency admission history.

**Fig. 13:**
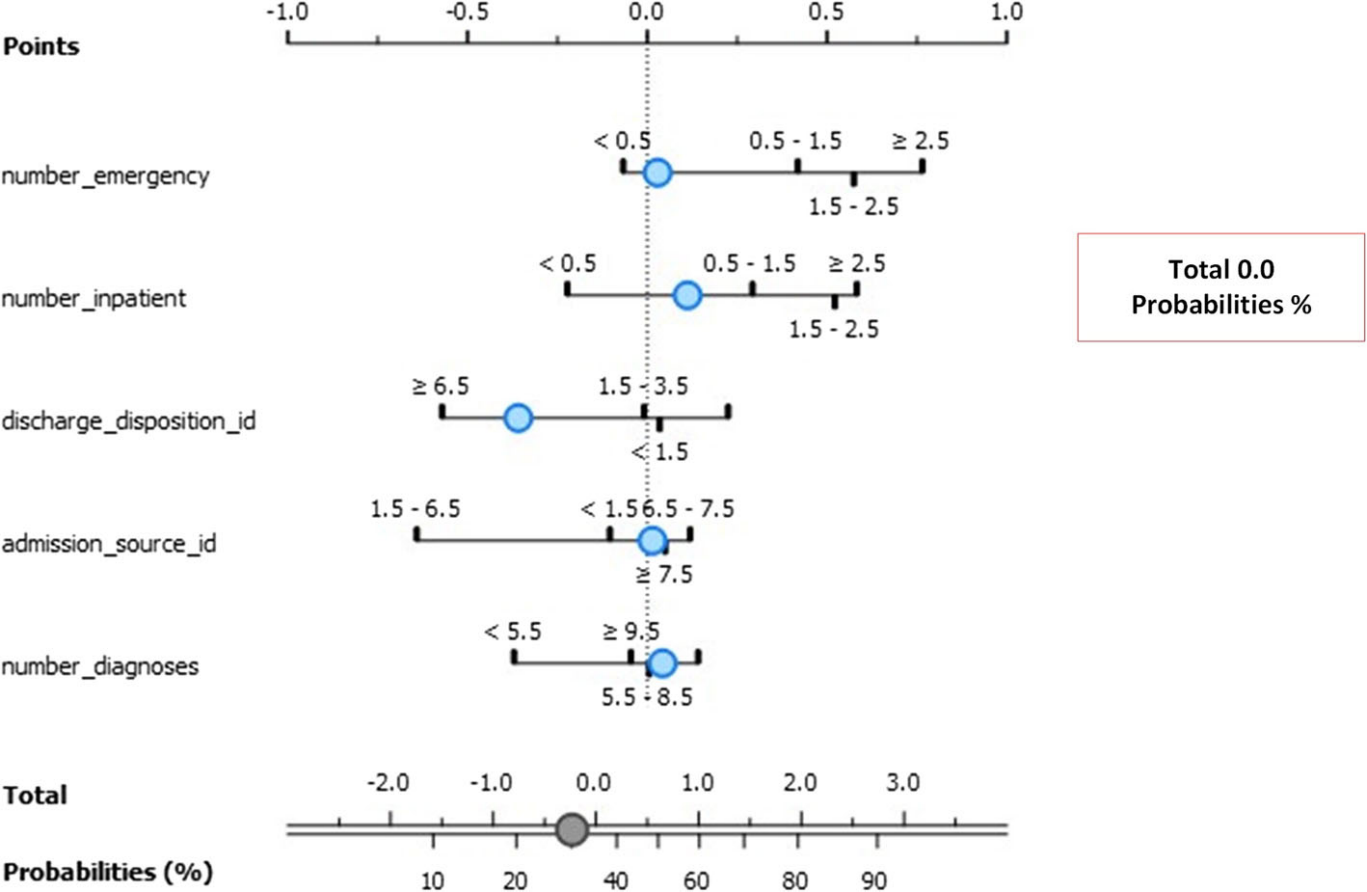
Nomogram visualization of Naïve Bayes classifier on target class 2

Figure 13 depicts the exposure factors with absolute importance on Class 2 including the number of emergencies, the number of patients, discharge disposition ID, admission source ID, and number of diagnoses. The log odds ratios illustrate the association between these exposure factors to lack of re-admission after discharge. The conditional probability for re-admission after discharge based on these exposure factors is 0.42. The number of emergency admission increases re-admission chances by 80% for those with least history. Further, those with higher inpatient admission history have 65% chance of re-admission for more than 30 days. Most importantly, patients who undergo more than 9.5 diagnoses tests have 70% chance of re-admission for more than 30 days after discharge.

## Conclusion

The size of the health data and the amount of information contained exemplifies the importance of machine learning in the health sector. Developing the profiles for the patients can help in understanding the factors that help reduce the burden of the disease while at the same time improve outcomes. Diabetes is a major problem given that over 78% of the patients admitted across the 130 hospitals were treated for the condition. Of the total number of diabetic patients who participated in the study, over 47% were readmitted with over 36% percent staying in the hospital for over 30 days. This study has also established that women and Caucasians are more vulnerable to hospital re-admissions [5, 33–39]. Each of the machine learning models has established different combinations of features influencing the admission rates. For instance, LDA proposes a linear combination while the SVM suggests a third-degree polynomial degree of association between re-admission and its predictors. Further, J48 models the relationship as non-linear with emphasis on the importance of emergency admission and in-patient treatment on re-admission rates. kNN models lead to the conclusion that fewer number of lab procedures, diagnoses, and medications lead to increased higher re-admission rates. Diabetic patients who do not undergo vigorous lab assessments, diagnosis, medications are more likely to be readmitted when discharged without improvements and without receiving insulin administration, especially if they are women, Caucasians, or both.

## Supplementary information

**Additional file 1** List of features and descriptions in the experiment datasets.

## Abbreviations

AI: Artificial intelligence
ANOVA: Analysis of variance
C4.5: Data mining algorithm
DA: Discriminant analysis
HbA1c: Glycated hemoglobin test
ID3: Iterative dichotomiser 3
J48: Decision tree J48
KNN: K-nearest neighbors
LA: Linear discriminant
ML: Machine learning
NB: Naive Bayes
Non-ICU: Non intensive care unit
RF: Random forest
ROC: Receiver operating characteristic
SVM: Support vector machine
