# Correction to: Implementation of machine learning algorithms to create diabetic patient re-admission profiles

**DOI:** 10.1186/s12911-020-1102-7

**Published:** 2020-05-18

**Authors:** Mohamed Alloghani, Ahmed Aljaaf, Abir Hussain, Thar Baker, Jamila Mustafina, Dhiya Al-Jumeily, Mohammed Khalaf

**Affiliations:** 1 Artificial Intelligence Office, Dubai, UAE; 2 Liverpool John Moores University, Computer science Department, Liverpool, UK; 3 The University of Anbar, Al-Tameem Street, Al-Anbar, Al-Ramadi, 55431 Iraq; 4 Kazan Federal University, Kremlyovskaya St, 420008 Kazan, Republic of Tatarstan Russia; 5 Department of Computer Science, Al-Maarif University College, Anbar, The city of Ramadi, 31001 Iraq

**Correction to: BMC Med Inform Decis Mak 19, 253 (2019)**

**https://doi.org/10.1186/s12911-019-0990-x**

In the publication of this article [1], there were two errors.

The error: Authors Ahmed Aljaaf^1,3^, Abir Hussain^1^, Thar Baker^1^, and Dhiya Al-Jumeily^1^ are affiliated with affiliation 1.

It should instead read: Ahmed Aljaaf^2,3^, Abir Hussain^2^, Thar Baker^2^, and Dhiya Al-Jumeily^2^ since the authors should instead be affiliated with affiliation 2: Computer science Department, Liverpool John Moores University, Liverpool, UK.

The error: the Acknowledgements section lacks additional acknowledgements.

It should instead read:

**Acknowledgments**

Dr. Mohamed Alloghani is an Advisor for the Minister of State for Artificial Intelligence in the United Arab Emirates and working at the Artificial Intelligence Office, Dubai. The data sources used in the paper was retrieved from UCI Machine Learning Repository as submitted by the Center for Clinical and Translational Research. The organization and the characteristics of the data made it easy to complete classification and clustering tasks. The authors would like to thank the eSystems Engineering Society for the support provided to the work presented in this paper.

This has now been updated in this correction article.
