# Supplementary figures and images for: Implementation of machine learning algorithms to create diabetic patient re-admission profiles
Source: BMC Med Inform Decis Mak. 2019 Dec 12;19(Suppl 9):253. doi: 10.1186/s12911-019-0990-x (PMC6907102; doi:10.1186/s12911-019-0990-x)

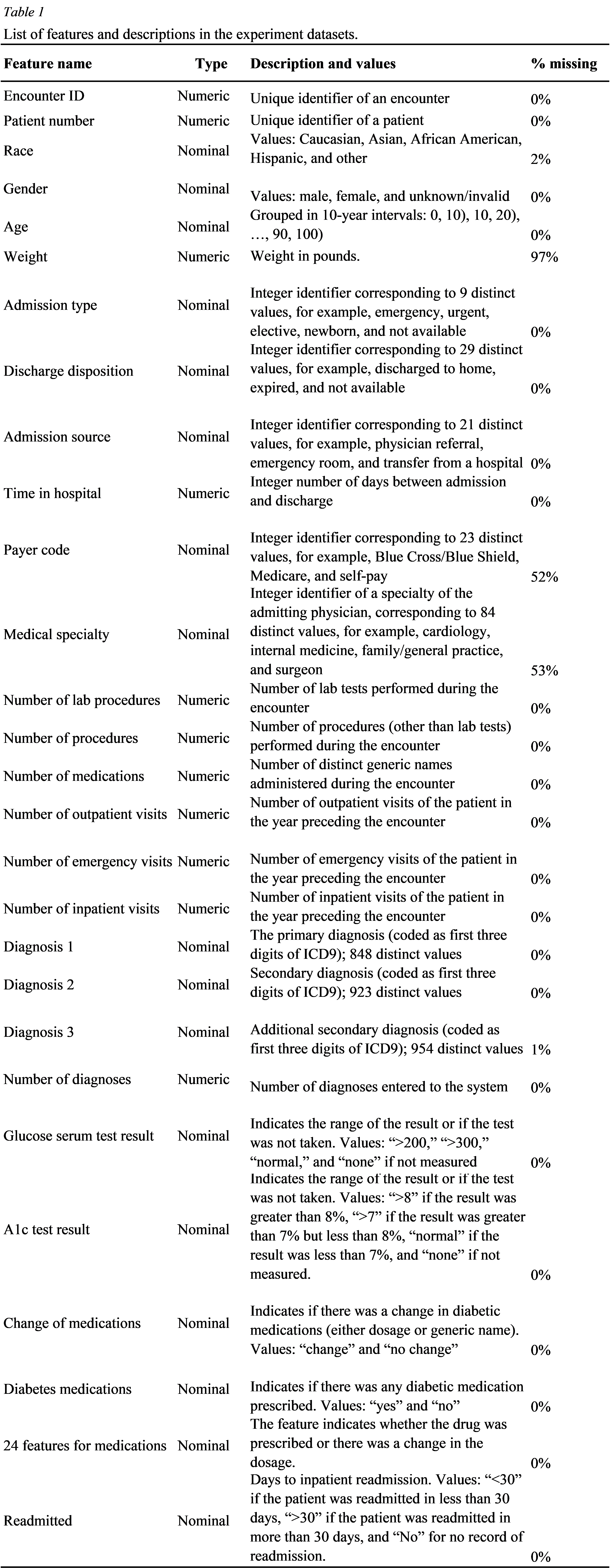

Supplement: Supplementary file 1 — Additional file 1 List of features and descriptions in the experiment datasets. [file 12911_2019_990_MOESM1_ESM.png]
